# Impact of oral *Chlamydia* vaccination on host gut microbiome and metabolite composition

**DOI:** 10.1128/msystems.01285-25

**Published:** 2025-11-10

**Authors:** Youyou Huang, Jiao Wan, Chuqiang Shu, Xichun Yan, Jingyue Ma, Tian Zhang, Jiarong He, Ziqing Wan, Guang Li, Qi Zhang, Zengzi Zhou, Xin Sun, Jing Zhao, Pu Zhang, Luying Wang, Tianyuan Zhang, Qi Tian

**Affiliations:** 1 Department of Obstetrics and Gynecology, The Affiliated Maternal and Child Health Care Hospital, Hengyang Medical School, University of South China (Hunan Provincial Maternal and Child Health Care Hospital) 117925 , Changsha, China; 2 National Health Commission Key Laboratory of Birth Defect for Research and Prevention (Hunan Provincial Maternal and Child Health Care Hospital), Changsha, Hunan, China; 3 Department of Dermatovenereology, Tianjin Medical University General Hospital/Tianjin Institute of Sexually Transmitted Disease, Tianjin, China; 4 Department of Obstetrics and Gynecology, The Third Xiangya Hospital, Central South University, Changsha, Hunan, China; 5 Fujian Provincial Maternal and Child Health Hospital, Fuzhou, Fujian, China; 6 Shanghai Institute of Virology, Shanghai Jiao Tong University School of Medicine, Shanghai, China; Pacific Northwest National Laboratory, Richland, Washington, USA

**Keywords:** *Chlamydia*, oral vaccines, genital pathology, gut microbiome, gut metabolites

## Abstract

*Chlamydia trachomatis*, an intracellular pathogen, is recognized as the most common sexually transmitted bacterial infection among women worldwide. *Chlamydia* infections can lead to undesirable clinical outcomes, including pelvic inflammatory disease and infertility. Recently, the gut has been identified as a niche for *Chlamydia* colonization in human gut-derived organoids. However, despite the biological impact on the host remaining under investigation, oral inoculation of *Chlamydia* as a whole-organism vaccine has been reported as a promising strategy for preventing genital *Chlamydia* infections in mice. Few studies have evaluated the impact of oral *Chlamydia* vaccination on the gut microbiome and metabolite changes. In this study, we assessed time-series alterations in the gut microbiome and metabolites following oral *Chlamydia muridarum* inoculation in a mice model, and we analyzed the composition and correlation between serum immune parameters and the sequencing profiles in the host. We identified 129 microbial changes and 186 differentially abundant metabolites in the gut across various vaccination approaches during the 30-day immunization process. Additionally, we discussed the potential influence of live *Chlamydia* on gut epithelium and the biomarkers of effective immunization based on correlation analysis.

**IMPORTANCE:**

*Chlamydia* infections primarily lead to morbidity rather than mortality. Consequently, in developing and implementing a *Chlamydia* vaccine, the utmost priority is evaluation of safety. As a promising yet controversial approach, live oral vaccination for *Chlamydia* raises concerns regarding its impact on the host’s gut environment. Our study not only investigates changes in the gut microbiome and metabolites during vaccination but also identifies changes in gut epithelium during vaccination and potential biomarkers during immunization. These findings are crucial for the development of whole-organism oral *Chlamydia* vaccines and provide valuable insights into the long-term colonization of *Chlamydia* in the gut.

## INTRODUCTION

*Chlamydia trachomatis* is the most common sexually transmitted infection globally, and it can lead to serious complications in the female upper genital tract, such as pelvic inflammatory disease and infertility (1). Although antibiotics can effectively treat the infection, the asymptomatic nature of infection poses challenges for prevention and timely intervention (2). In this context, vaccination could play a crucial role in preventing *C. trachomatis* infections and offering a long-term solution to this challenge (3). Currently, there are few licensed *Chlamydia* vaccines available for human use (4). Among the various types of vaccines, whole-organism *Chlamydia* vaccines have shown effective protection in animal studies (5). In 2015, a deactivated form of *C. trachomatis* was modified and used to induce protective immunity in preclinical studies (6). Additionally, live-attenuated vaccines derived from *Chlamydia psittaci* have been approved for use in poultry to protect against avian chlamydiosis, and vaccines derived from *Chlamydia abortus* are licensed for sheep and goats to prevent ovine enzootic abortion (7, 8).

Oral inoculation of live *Chlamydia muridarum* provides strong protection in mice. This leads to a shorter duration of *Chlamydia* reinfection and less pathology in the upper genital tract (9). Even though gut *C. muridarum* vaccination is effective in the mice, the effect of the vaccine on the host still needs to be carefully studied. In nature, *Chlamydia* is a genital pathogen that is often found in the gastrointestinal (GI) tracts of various hosts, including humans (10, 11). The gut may serve as a natural niche for *Chlamydia* colonization, and some findings suggest that gut *Chlamydia* co-infection with genital *Chlamydia* during the acute phase could potentially trigger pathogenic immune responses in the upper genital tract (12, 13). The mechanism of how *Chlamydia* gastrointestinal infection occurs remains to be studied in humans. Women’s rectal *C. trachomatis* infections may arise from vaginal autoinoculation. Batteiger et al. (14) proposed an alternative hypothesis suggesting that oral sex could also lead to gastrointestinal infections, since oral exposure to *Chlamydia* can result in positive gastrointestinal infections in men, where autoinoculation is less likely.

Research has explored the possibility of using inactivated *Chlamydia* as a vaccine. This approach aims to provide protective immunity while minimizing the risks linked to live *Chlamydia*. However, findings suggest that inactivated *Chlamydia* may offer inferior protective immunity relative to live *Chlamydia*. This difference might be due to the ability of live *Chlamydia* to grow and replicate, which increases the variety of antigens presented to the immune system (4). However, the exact reasons why live *Chlamydia* produces stronger protective immunity than killed organisms are still unknown.

Microbes and their metabolites could be crucial factors influencing the effectiveness of a mucosal *Chlamydia* vaccine. For example, commensal microbes and their metabolites can regulate tissue-resident memory T (Trm) cells through mechanisms such as shaping local cytokine environments, modulating antigen presentation, and influencing metabolic fitness. These processes support the formation and maintenance of protective Trm cell responses, which are particularly important for long-term immunity against *C. trachomatis* (6, 15).

This underscores the importance of studying the gut microbiome, metabolite profiles, and the impact of vaccination on the gut epithelium during the immunization process. In this study, we examined the changes in gut epithelial cells, microbial populations, and metabolite profiles over time after oral inoculation with live or inactivated *Chlamydia*. We also assessed the resulting immune responses and investigated the correlations between microbial dynamics and metabolite levels following immunization. This information can provide insights into how gut microbes and their metabolic products affect the effectiveness of immune responses, potentially aiding in the development of more effective vaccine strategies.

## RESULTS

### Oral inoculation with live *Chlamydia* results in sustained gut colonization and induces protective immune response

**Fig 1:**
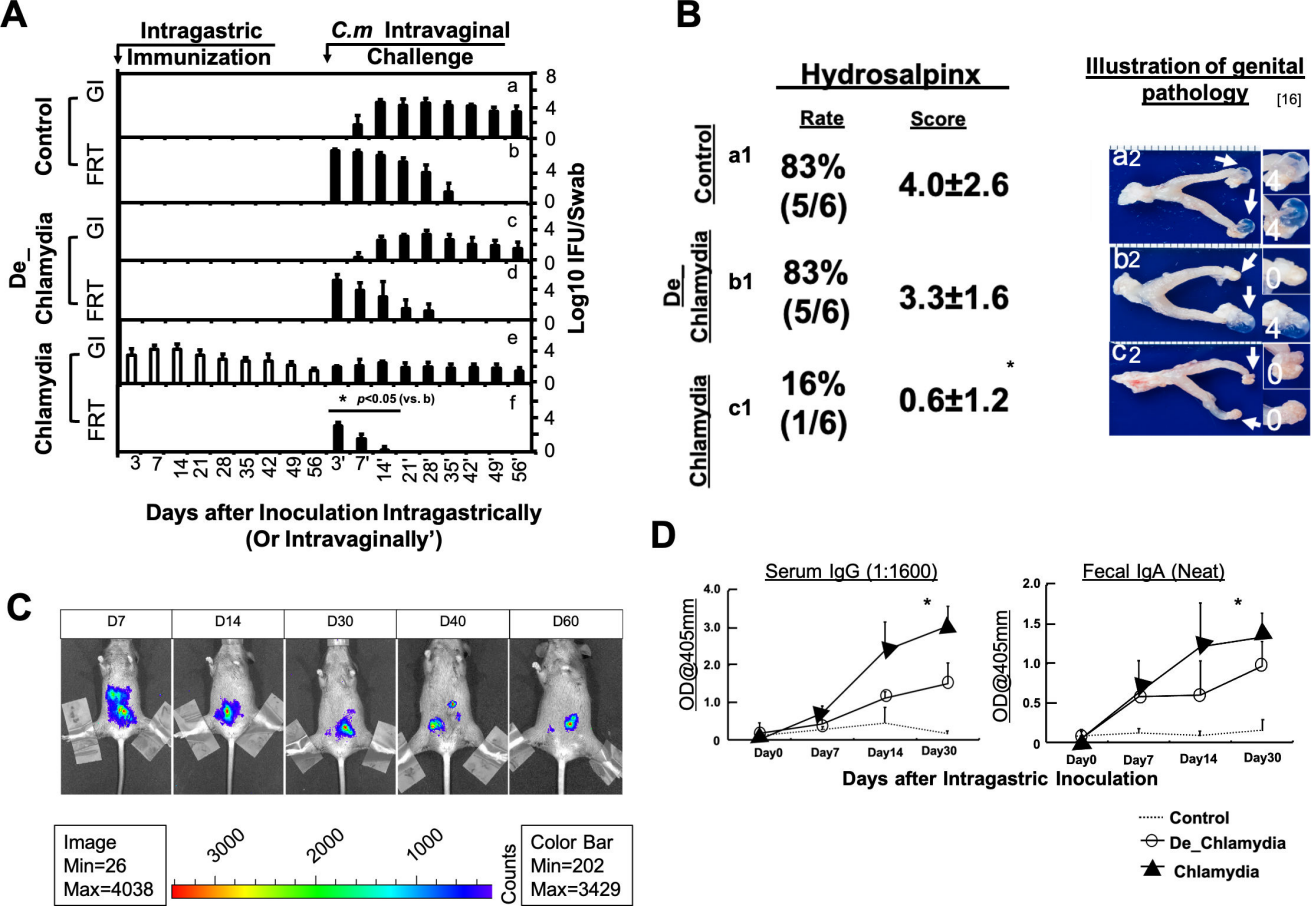
Oral administration of live *Chlamydia* leads to a long-lasting gut infection and exerts immune effects against genital pathology. Mice were intragastrically inoculated with SPG buffer (control, *n* = 6), 2 × 10^5^ IFU heat inactivated *C. muridarum* (De_*Chlamydia*, *n* = 6), or 2 × 10^5^ IFU live *C. muridarum* (*Chlamydia*, *n* = 6) and challenged intravaginally with 2 × 10^5^ IFU live *C. muridarum* on day 60. (**A**) Shedding of *C. muridarum* from the gastrointestinal and female reproductive tracts is shown as log10 IFU per swab; only significant differences are labeled (*P < 0.05, Wilcoxon rank-sum test). No significant difference was observed between the heat-inactivated *Chlamydia* group and the control group. Panels a2 and c2 are adapted from a previous publication with permission (16). Panel b2 is from the same batch of mice described in reference 17 but was not included as a representative image in that publication. (**B**) Upper genital tract pathology assessed on day 60. Representative macroscopic images of an entire genital tract from a previously published team paper (16) were given as an illustration to genital pathology (a2, b2, and c2); incidence and severity were quantified (*P < 0.05, Fisher’s exact test or Wilcoxon rank-sum test). (**C**) Bioluminescence imaging of luciferase-expressing *C. muridarum* illustrates colonization over time (signal intensity: red > green > blue). (**D**) Fecal IgA and serum IgG levels measured by ELISA (*P < 0.05, Wilcoxon rank-sum test; only significant differences were labeled).

Three groups of female C57BL/6J mice were intragastrically inoculated with live *Chlamydia* (*n* = 6), heat-inactivated *Chlamydia* (labeled as De_*Chlamydia* in the figures, *n* = 6), and a vehicle control (sucrose phosphate glutamate [SPG] buffer, *n* = 6), respectively. The deactivation of the *Chlamydia* was accomplished through heat treatment and was confirmed by a cell infection assay, which indicated that no viable elementary body (EB) remained. In detail, mice were intragastrically inoculated with SPG buffer only (control group, *n* = 6) (Fig. 1A, panels a and b), 2 × 10^5^ inclusion forming units (IFU) of deactivated *C. muridarum* (immunization group 1, *n* = 6) (Fig. 1A, panels b and c), or 2 × 10^5^ IFU of live *C. muridarum* (immunization group 2, *n* = 6) (Fig. 1A, panels c and d). These mice were challenged intravaginally on day 60 post-immunization with 2 × 10^5^ IFU of *C. muridarum*. Results are presented as the log10 number of IFU per swab specimen on the *y*-axis. The infection burden was monitored by calculating *Chlamydia* IFUs recovered from genital and rectal swabs. After inoculation, live *Chlamydia* oral inoculation resulted in persistent *Chlamydial* colonization in the gut, and live *Chlamydia* was detected in rectal swabs even after 50 days post-inoculation. Notably, no *Chlamydia* was recovered from genital swabs, indicating no cross-contamination from the gut to the genital tract after vaccination (Fig. 1A, panels e and f). The persistence of *Chlamydia* in the gut after immunization could also be monitored using *in vivo* imaging with luciferin-labeled *Chlamydia*, with live *Chlamydia* detectable up to day 60, indicating sustained gut colonization. (Fig. 1C).

Sixty days after immunization, the mice were intravaginally challenged with 2 × 10^5^ IFU of wild-type *C. muridarum* in all three groups. The genital infection burden was significantly lower in the live *Chlamydia* group compared to the deactivated *Chlamydia* group (Fig. 1A, panel f vs d), demonstrating a more protective effect against genital mucosal *Chlamydia* infection. The duration of genital infection was shorter in both the live *Chlamydia* group and the deactivated *Chlamydia* group compared to the control group, suggesting that the vaccination provided mucosal protection (Fig. 1A, panels f and d vs b). Additionally, mice were sacrificed on day 60 following the intravaginal challenge to evaluate upper genital tract pathology. The assessment of genital pathology at 60 days post-challenge revealed a significant reduction in hydrosalpinx in the live *Chlamydia* group compared to the other two groups.

Mice immunized with live *Chlamydia* in the GI tract showed a significantly lower incidence (*P* < 0.05, Fisher’s exact test) and reduced pathology scores (*P < 0.05, Wilcoxon rank-sum test) when compared to control mice. No significant changes were observed between the deactivated *Chlamydia* and control groups. These data suggest that while deactivated *Chlamydia* immunization offers certain, though not significant, levels of protection against mucosal infection, it is less effective in preventing genital pathology outcomes. Similarly, enzyme-linked immunosorbent assay (ELISA) results showed that serum IgG and fecal IgA antibodies against whole EBs were significantly higher in the live *Chlamydia* group than in the deactivated *Chlamydia* and control groups (Fig. 1D).

### *Chlamydia* colonization temporarily alters the ultramicroscopic structure of the colon epithelium

To evaluate the effects of immunization on colon epithelial cells, a separate cohort of mice was utilized. In this part, mice were only immunized accordingly without challenge. On days 7, 14, 30, and 60 post-immunization, three mice from each group were sacrificed to assess the effects of immunization on colon epithelial cells. Both hematoxylin and eosin (H&E) staining and transmission electron microscopy (TEM) were performed.

**Fig 2:**
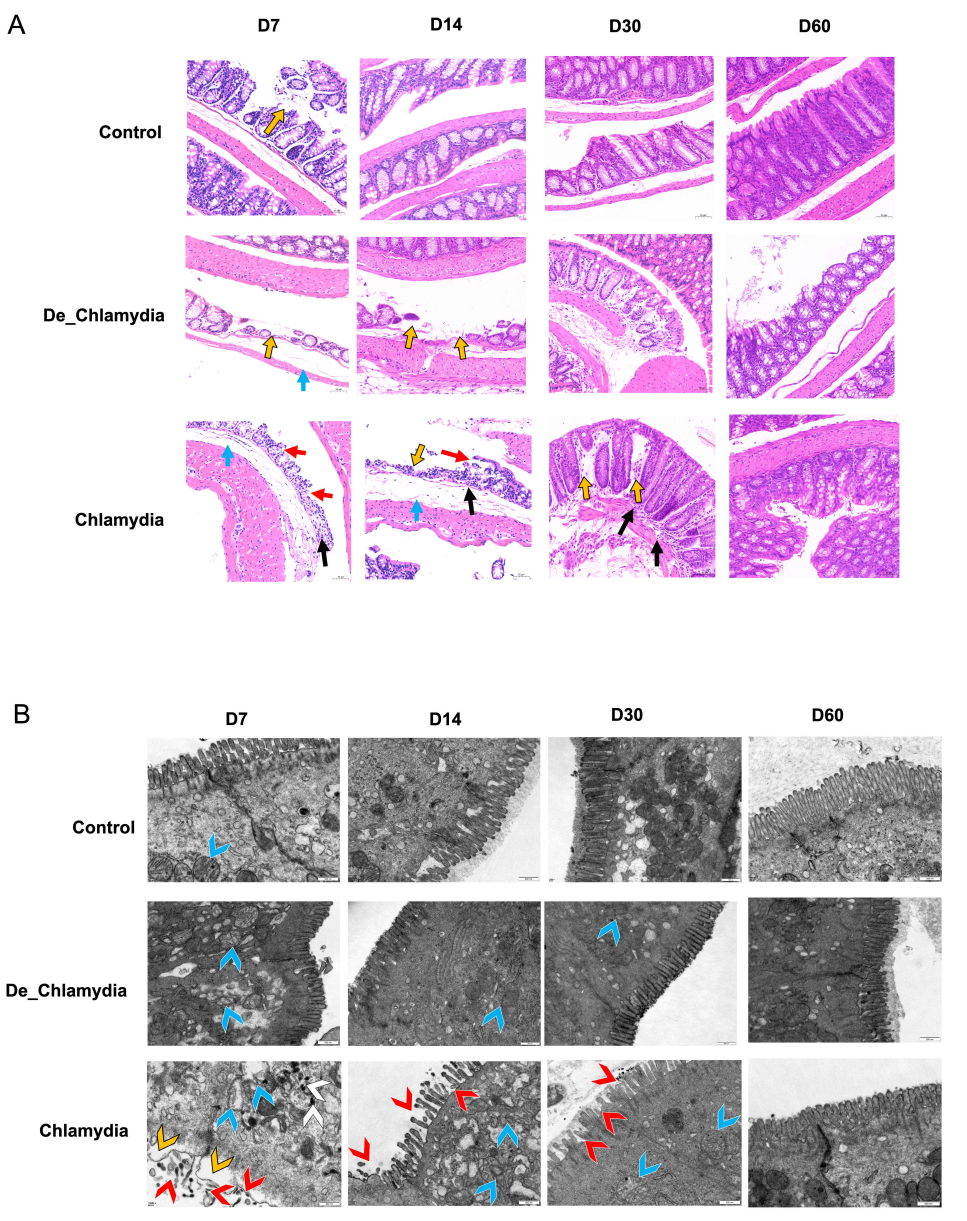
Ultrastructural analysis of colon epithelial cells following immunization. Hematoxylin and eosin-stained and transmission electron microscopy (TEM) images reveal changes in microvilli integrity and mitochondrial morphology of colon tissue among control, live, and deactivated *Chlamydia* treatment groups. (**A**) Hematoxylin and eosin-stained colon tissues. Observations include submucosal edema (blue arrows) and shedding of the mucosal crypt structures (yellow arrows). Local shedding of the mucosal epithelial layer is evident, with exposure of the underlying lamina propria (red arrows). In addition, inflammatory cell infiltration is indicated (black arrow), along with further shedding of the mucosal crypts (yellow arrows). (**B**) TEM of colon tissues. TEM images highlight variations such as villi structure shedding (red arrowheads) and irregular morphology, vacuolization in the cytoplasm with decreased electron density (yellow arrowheads), swollen mitochondria with reduced matrix electron density (blue arrowheads), and the presence of autophagosomes (white arrowheads).

Hematoxylin and eosin-stained colon tissues (Fig. 2A) showed that in the control group, at all-time points, the mucosal epithelial cells were arranged in a neat and compact pattern. There was no obvious cell shedding or necrosis; the architecture of the mucosal glandular structures (crypts) was intact; and no significant inflammatory cell infiltration was observed. Furthermore, in the De_ *Chlamydia* group, on day 7, submucosal edema was noted (blue arrow); on days 7 and 14, partial shedding of the mucosal crypt structures was observed (yellow arrow). By days 30 and 60, the colon tissue appeared normal. In the *Chlamydia* group, on days 7 and 14, local shedding of the mucosal epithelial layer was observed with exposure of the underlying lamina propria (red arrow), along with submucosal edema (blue arrow). In addition, inflammatory cell infiltration was visible on days 7, 14, and 30 (black arrow), and partial shedding of the mucosal crypts was noted (yellow arrow). By day 60, the colon tissue appeared normal.

TEM of colon tissues (Fig. 2B) revealed that in the *Chlamydia* group at day 7, compared with the control group, the colonic epithelial villi displayed shedding, reduced density, and irregular morphology (red arrowhead). Local regions within the cytoplasm showed vacuolization with decreased electron density (yellow arrowheads); mitochondria were swollen with reduced matrix electron density (blue arrowheads), and autophagosomes were visible within the cytoplasm (white arrowheads). Improvements in these features were observed on days 14 and 30 compared to day 7. By day 60, the colon epithelium appeared normal. Overall, the alterations in the epithelial structure after *Chlamydia* inoculate were most pronounced on days 7, 14, and 30, with the villus morphology returning to normal appearance by day 60.

### Time-series analysis of bacterial community changes between different vaccinations

In order to look for the different impacts of introducing live vs deactivated organisms on gut and find potential factors that may influence the efficacy of vaccination, we evaluated the bacterial community changes at different time points through the α-diversity and β-diversity between different groups. The microbial community composition was analyzed using 16S rRNA gene sequencing, targeting the V3–V4 variable regions. Bacterial community changes were evaluated in all three groups. Fecal samples were taken at four time points after immunization: days 0, 7, 14, and 30. DNA was extracted from the fecal samples, and libraries were constructed and sequenced. A total of 63 fecal samples were sequenced, yielding an average of 54,360 ± 9,165 reads per sample. QIIME2 was used to map reads to amplicon sequence variants (ASVs), which were then clustered to determine the microbial community composition.

**Fig 3:**
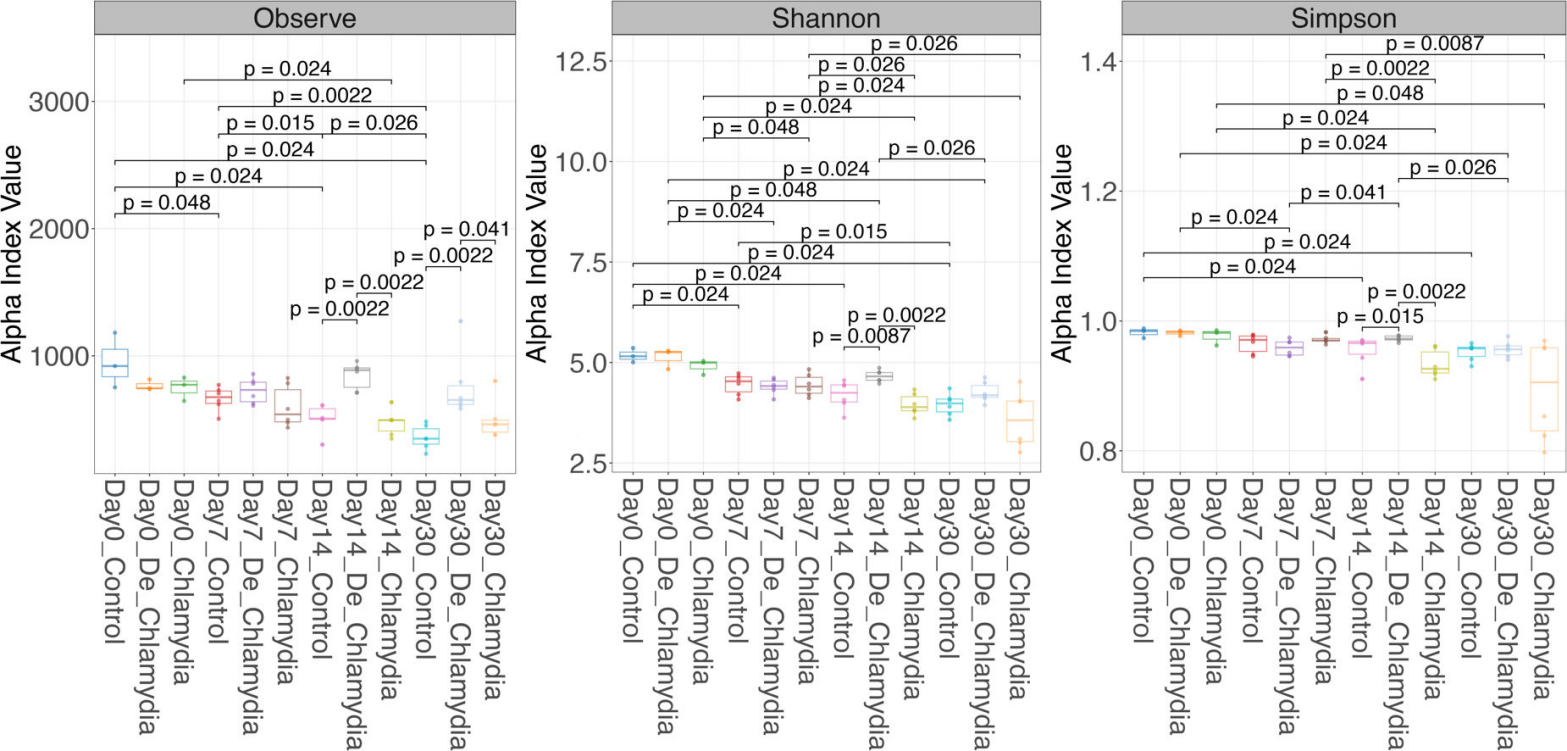
Microbial α-diversity changes dynamically in microbial communities after immunization. The α-diversity indices across groups at different time points, evaluating observed species, Shannon index, Simpson index. Only *P* values less than 0.05 are indicated, as determined by the Wilcoxon rank-sum test.

The α-diversity indices, including observed species, Shannon, and Simpson indices (Fig. 3), were utilized to evaluate the diversity and richness of the GI flora after inoculations. These indices decreased in both the control and *Chlamydia* groups over time, whereas the heat-inactivated *Chlamydia* group exhibited an increase on day 14. At each time point, the indices were similar between the control and *Chlamydia* groups, while the heat-inactivated *Chlamydia* group demonstrated higher indices on days 14 and 30. The quality control of the sequencing and dynamic changes in phyla and genus-level flora abundance over time, both in grouped and individual samples, is presented in the supplementary figures (Fig. S1 and S2). The dynamic changes in phyla and genus-level flora abundance over time, both in grouped and individual samples, showed that the dominant phyla included *Bacteroidetes*, *Firmicutes*, *Proteobacteria*, and *Verrucomicrobia*, with fluctuations in their abundance observed over time and differing between groups (Fig. S2). The dominant genus identified included *S24-7*, *Prevotella*, *Lactobacillus*, *Akkermansia*, *Lachnospiraceae*, and *Clostridiales*, along with other genera. To further investigate the impact of oral *Chlamydia* vaccination on the gut flora community, β-diversity analysis was conducted. On days 14 and 30, the β-diversity of the heat-inactivated *Chlamydia* group was significantly different from that of the control and *Chlamydia* groups (Fig. S3).

**Fig 4:**
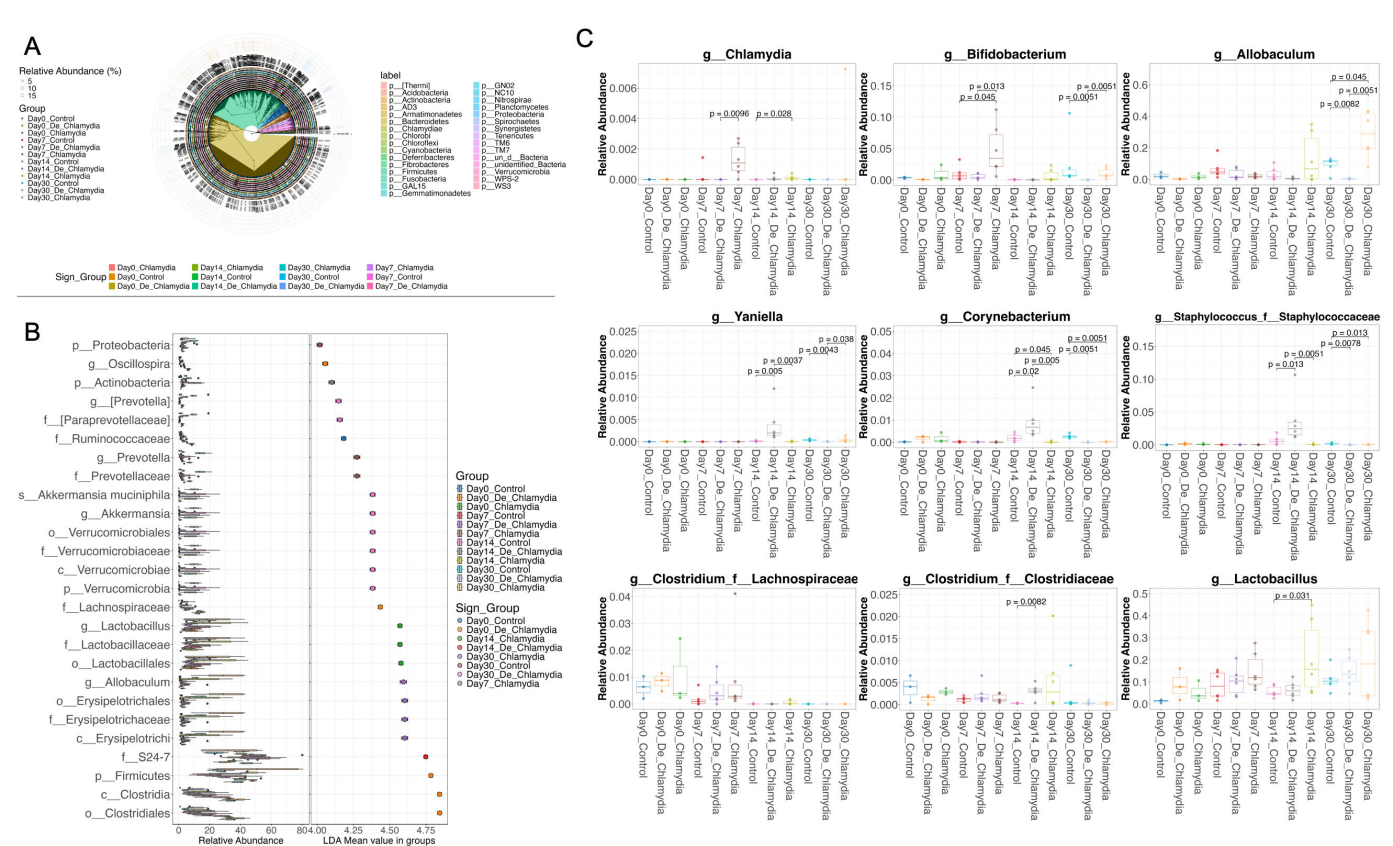
LEfSe analysis identifying differentially abundant taxa in the gut microbiome during vaccination. (**A**) Group LEfSe results through a microbial phylogenetic graph (Kruskal–Wallis test, *P* values adjusted with the false discovery rate, and post hoc analysis performed with the Wilcoxon test, applying a cutoff of *P* < 0.05). (**B**) Microbes with linear discriminant analysis (LDA) scores greater than 4 (for those with LDA >2, see Fig. S4). (**C**) Representative microbes were identified, and significant changes in microbial taxa abundance at the genus level over time are noted (*P < 0.05, Wilcoxon test).

Additionally, we identified major alterations in gastrointestinal bacterial taxa through sample and group linear discriminant analysis effect size (LefSe) analysis (Fig. 4A and B), and detected 129 significantly changed abundances of GI taxa over the time series (linear discriminant analysis [LDA] score >2.0, Fig. S4). Notably, at the genus level, *Bifidobacterium* and *Chlamydia* were enriched in the *Chlamydia* group on day 7. The genera *Allobaculum*, *Lactobacillus*, and g_*Clostridium_*f_*Clostridiaceae* showed increased abundance in the *Chlamydia* group on days 14 and 30. In the heat-inactivated *Chlamydia* group, a rise in the abundance of genera *Yaniella*, *Corynebacterium*, and *Staphylococcus* was also observed on day 14. Across all three groups, g_*Clostridium_*f_*Lachnospiraceae* exhibited a decreasing trend over the time series, while *Lactobacillus* demonstrated an increasing trend (Fig. 4C).

### Unique metabolic alterations induced by live *Chlamydia* on day 7

**Fig 5:**
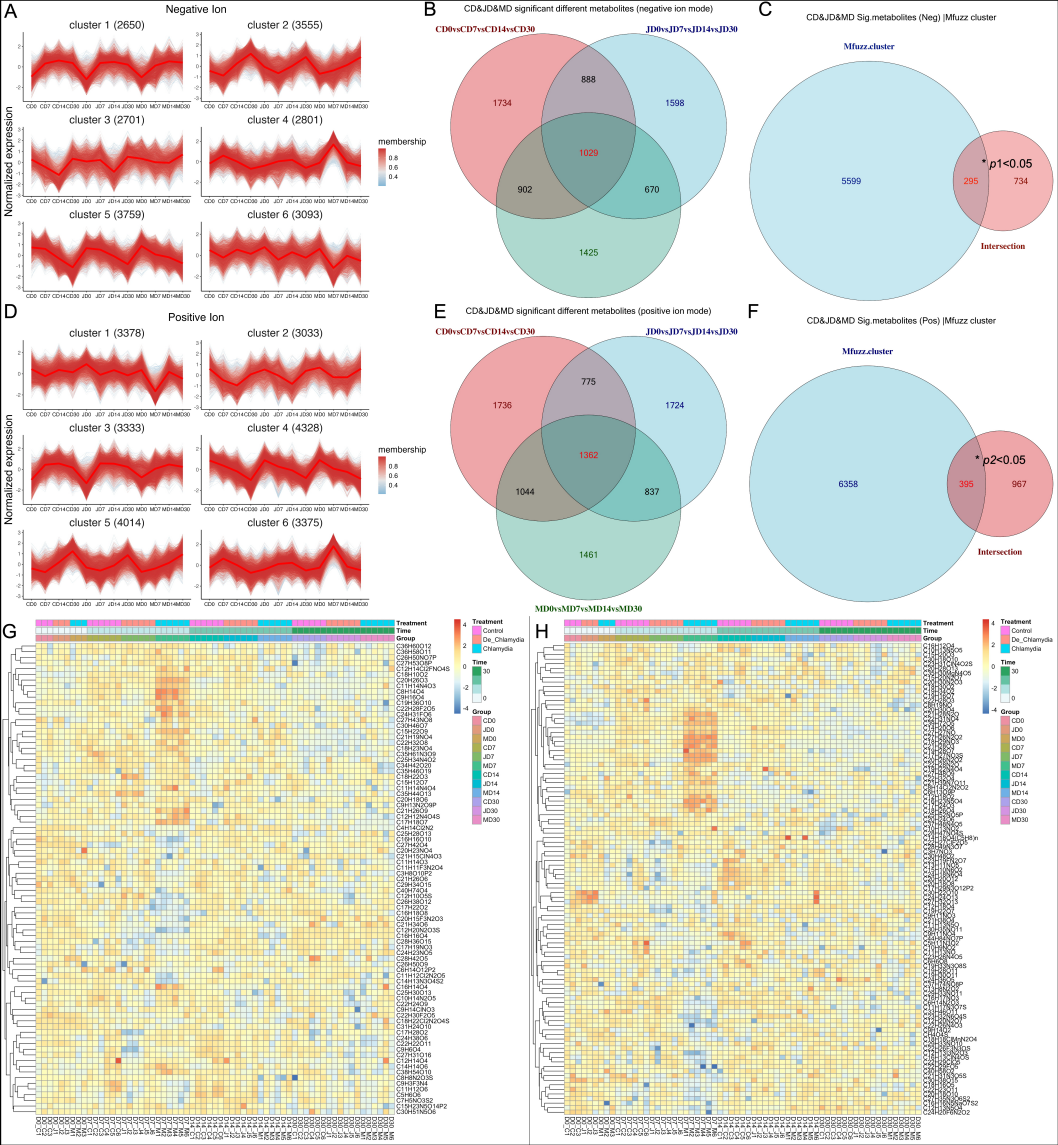
LC-MS/MS analysis of metabolite changes in response to oral *Chlamydia* vaccination. Differentially abundant metabolites identified in negative and positive ion modes are outlined, with clusters illustrating metabolite abundance trends over time. Fecal samples were collected at different time points (D0, D7, D14, and D30) alongside feces for 16S rRNA sequencing, and the fecal samples were subjected to LC-MS/MS analysis. (**A**) The metabolomic profiles from various time points and groups were analyzed using the Mfuzz method. This analysis aimed to identify clusters of metabolite patterns across different samples and to establish relationships between time and metabolites within these clusters. Notably, on day 7 (clusters 4 and 6), the live *Chlamydia* group exhibited the most significant metabolic changes among all groups and time points. (**B**) We focused on the differential metabolites produced at each time point after each treatment, then considered the intersections of three different treatments to identify biomarkers that may vary with time across each condition. The analysis of differential metabolites included comparisons: CD0 vs CD7 vs CD14 vs CD30, DD0 vs DD7 vs DD14 vs DD30, and LD0 vs LD7 vs LD14 vs LD30 (Table S2). Here, C, D, and L denote samples from the control, deactivated *Chlamydia*, and live *Chlamydia* groups, respectively, while the number indicates the sampling day (e.g., CD0 represents the control group at day 0). An intersection of the differentially abundant metabolites yielded 1,029 metabolites. (**C**) Metabolites from Mfuzz clusters (clusters 4 and 6). From these 1,029 substances, we identified 295 metabolites that exhibited significant changes over time and showed differences across groups with *p1 < 0.05. (**D, E, F**) Analysis of metabolites identified in positive ion mode. We identified 395 metabolites that exhibited significant changes over time and showed differences across groups with *p2 < 0.05. (**G**) Heat map of metabolite abundances over time in negative ion mode. (**H**) Heat map of metabolite abundances over time in positve ion mode.

To evaluate the influence of vaccination on host metabolites, metabolites were extracted from the same fecal pellets used for 16S rRNA sequencing: days 0, 7, 14, and 30, along with the 16S rRNA samples. The metabolites in the time series were obtained using liquid chromatography–tandem mass spectrometry (LC-MS/MS) analysis (Fig. 5). We analyzed the intersecting differences in metabolites among different groups over the time series, resulting in the preliminary identification of 1,029 differentially abundant metabolites in negative ion mode and 1,362 in positive ion mode. To account for the dynamic changes in metabolites over time, we established a time series for the abundances of metabolites of the identified characteristic peaks and the metabolomic profiles from various time points, and the groups were analyzed using the Mfuzz method, a soft clustering algorithm that groups metabolites based on similar abundance patterns over time. Clusters were generated by first standardizing the data and then applying a fuzzy c-means algorithm, which assigns metabolites to clusters with membership scores reflecting the degree of association with each cluster, allowing identification of temporal patterns and relationships between metabolites and time points.

Ultimately, we observed significant differences between clusters 4 and 6 in the negative ion mode and between clusters 1 and 6 in the positive ion mode, all of which were from the *Chlamydia* group on day 7. We extracted the data from these clusters for further analysis. By intersecting clusters from the Mfuzz analysis with the previously identified differentially abundant metabolites, we matched these metabolites with HMDB (17), MassBank (18), LipidMaps (19), mzCloud (20), KEGG, and in-house chemical database provided by the Suzhou PANOMIX Biomedical Tech Co., Ltd (Table S1). Eventually, 83 and 103 specific differentially abundant metabolites with defined chemical formulas were identified from the negative and positive ion modes, respectively. Then, KEGG pathway analysis of the differentially abundant metabolites with KEGG IDs was presented (Fig. S5). The analysis revealed significant alterations in glyoxylate/dicarboxylate, pyrimidine, purine, D-amino acid, and fructose/mannose metabolism, with purine metabolism exhibiting the highest functional impact (Impact = 0.5), suggesting a potential role of nucleotide synthesis pathway remodeling in the process (the top 5 *P* value enriched metabolic pathways were labeled in the plot).

### Potential associations among the GI microbiome, metabolites, and *Chlamydia*-specific antibody

**Fig 6:**
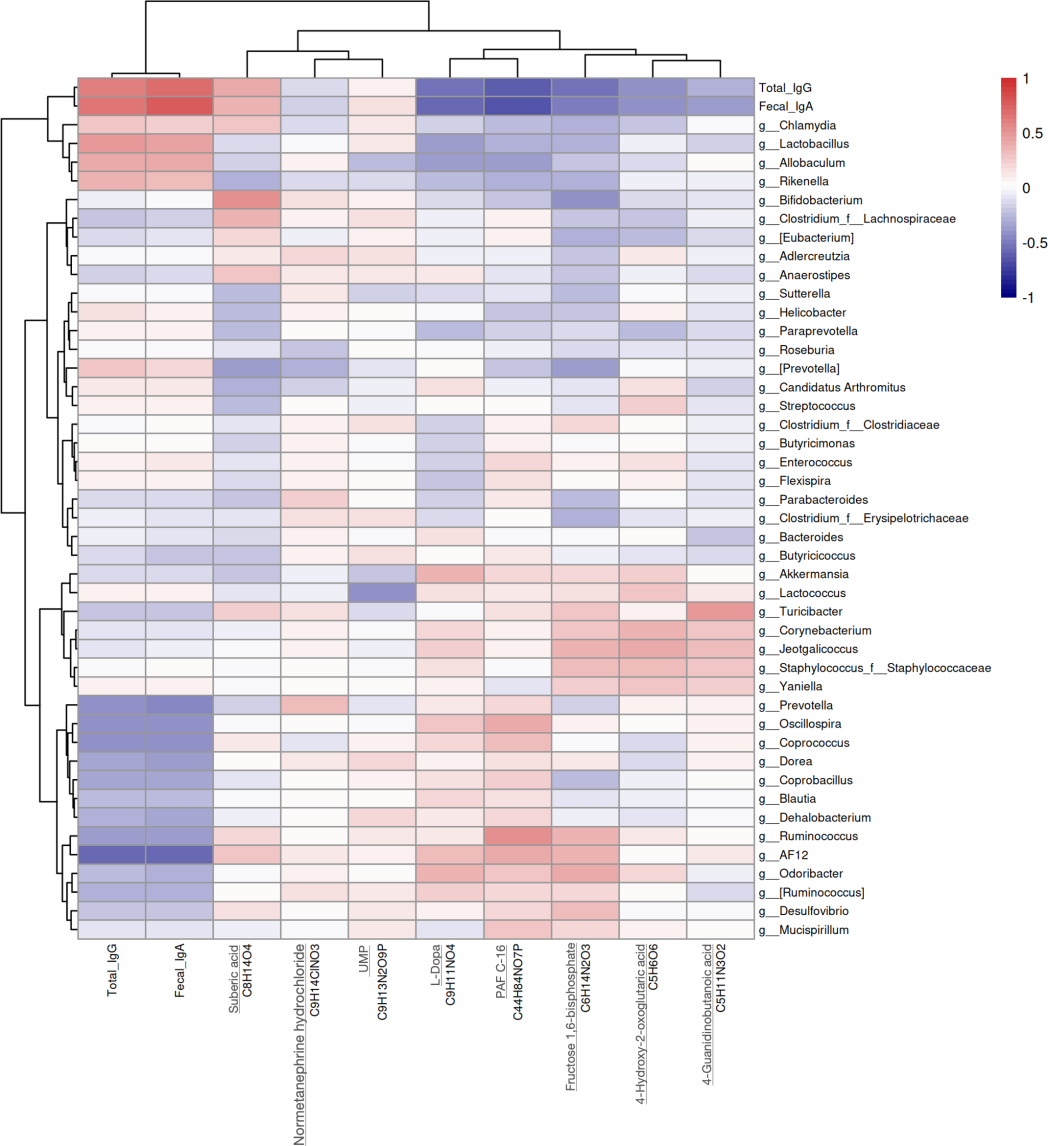
Pearson correlation analysis of the gut microbiome, metabolites, and *Chlamydia*-specific antibodies. The significant relationships among microbial taxa (at the genus level), metabolite levels, and antibodies. A total of 129 dominant microbial taxa (LDA >2) were analyzed in correlation with 186 differentially abundant metabolites identified from the non-targeted metabolomics approach (comprising 83 negative and 103 positive metabolites), as well as serum IgG and fecal IgA levels from different time points. Microbes at the genus level and metabolites that exhibit biological relevance are displayed.

We conducted a Pearson correlation analysis to explore the relationships between 129 predominant bacterial species from gut microbiome analysis (LDA >2), 186 differentially abundant metabolites identified through LC-MS/MS analysis (negative ion mode 83 and positive ion mode 103), and levels of *Chlamydia*-specific antibodies (serum IgG and fecal IgA) at various time points. This analysis enabled us to identify both positively and negatively correlated bacterial communities, metabolites, and antibodies with a significance level of *P* < 0.05 (Fig. 6).

There was a negative correlation between the production of antibodies (total IgG and fecal IgA) and the genus *AF12* (from the *Rikenellaceae* family), with Pearson values of −0.56 (*P* < 0.001) and −0.55 (*P* < 0.001), respectively. In addition to *AF12*, genera such as *Prevotella*, *Oscillospira*, *Coprococcus*, *Blautia*, *Dehalobacterium*, *Ruminococcus*, *Odoribacter*, and *Desulfovibrio* also displayed negative correlations with antibody levels. Furthermore, these antibodies were negatively associated with metabolites like levodopa (L-DOPA) and platelet activating factor C-16 (PAF C-16), with Pearson values ranging from −0.51 (*P* < 0.001) to −0.63 (*P* < 0.001). Conversely, genera including *Chlamydia*, *Lactobacillus*, *Allobaculum*, and *Rikenella* showed positive correlations with antibody production, exhibiting Pearson values between 0.25 (*P* = 0.040) and 0.48 (*P* < 0.001).

## DISCUSSION

Emerging evidence suggests that *Chlamydia* can enter the GI tract through various routes, including the oral route (13, 21). The process of *Chlamydia* spreading to and colonizing the GI tract occurs by steps (21, 22). Certain *C. muridarum* mutants display reduced capabilities in colonizing the GI tract (23, 24); for instance, mutants with no plasmid exhibit significant difficulties in colonizing the upper GI tract, whereas those deficient in specific chromosomal genes, such as tc0237 and tc0668, have more obstacles in the lower GI tract. This observation indicates that *Chlamydia* may depend on plasmids for its dissemination to the large intestine, while chromosome-encoded factors are necessary for sustaining colonization (25, 26).

Besides the various pathogenic virulence factors of *Chlamydia*, further research is needed to explore the interactions between *Chlamydia* and the host in the gut. This is especially important because gut microbiota and metabolites can significantly influence immune responses and the defensive mechanisms of gut epithelial tissues (27). However, such studies in the current literature are still limited. In the current study, we established a time-series evaluation following gut *C. muridarum* inoculation in mice, analyzing tissue structural, microbial, metabolic, and immune responses post-immunization. Our findings show that gut *Chlamydia* infection alters microbial and metabolite profiles and enhances antigen-specific antibody production.

*C. muridarum* infection in the murine genital tract is one of the most widely used preclinical models for examining chlamydial pathogenesis and assessing chlamydial vaccines (28–30). Following intravaginal inoculation, *C. muridarum* is known to lead to hydrosalpinx and infertility in mice, closely replicating the tubal adhesions observed in women with a history of *C. trachomatis* infection during laparoscopy (31, 32). In addition to infecting the mucosal surfaces of the mouse genital and respiratory tracts, *C. muridarum* can also inhabit the GI tract for prolonged periods without causing significant pathological changes in the genital region, making mice a suitable model for studying gut chlamydial vaccines (12).

The oral inoculation with *C. muridarum* as a vaccination strategy extends beyond mere research purposes (33). Gut-inoculated *C. muridarum* may induce cross-species immunity against human-type *Chlamydia* genital infections. Furthermore, mice previously exposed to *C. muridarum* have demonstrated heterotypic protection against subsequent infections with *C. trachomatis*. This suggests that the mouse-adapted *C. muridarum* could potentially protect humans from *C. trachomatis* infections (34). As a result, research teams are currently focused on developing *C. muridarum*-derived vaccines for human application (35, 36). Although murine and human microbiomes and metabolomes differ, our study in mice provides insights into the fundamental mechanisms of gut colonization, mucosal immune responses, and metabolic interactions following oral *Chlamydia* vaccination.

Our mouse model showed that live *Chlamydia* induced higher levels of *Chlamydia*-specific antibodies compared to inactivated *Chlamydia*. The mechanism for this observed phenotype remains unknown. This may relate to the ability to replicate in host. Live *Chlamydia* undergoes a distinctive biphasic developmental cycle within the host, and it includes a critical morphological transition from EBs to reticulate bodies, during which *Chlamydia* undergoes distinct changes in gene and protein expression (37). In contrast, heat-inactivated *Chlamydia* cannot undergo this transition and does not persist in the host like live EBs, and this could partially explain the lower levels of antibody production observed with heat-inactivated *Chlamydia*.

*Chlamydia* colonization in the gut is considered non-pathogenic, as researchers assessed through macroscopic evaluations and H&E staining 60 days after immunization (9, 38). Notably, in our study, by using multiple time-point evaluation, we found that gut *Chlamydia* inoculation temporarily alters the microscopic structure of the colon epithelium until day 30 post immunization (Fig. 2A and B). Specifically, we observed a reduction in the number of microvilli and swelling of mitochondria within the cytoplasm on days 7, 14, and 30. On day 60, the colon recovered from the *Chlamydia* inoculation. These findings indicate potential impacts of wild-type live *Chlamydia* as a vaccine on the gut, underscoring the necessity for further research in this area.

**Fig 7:**
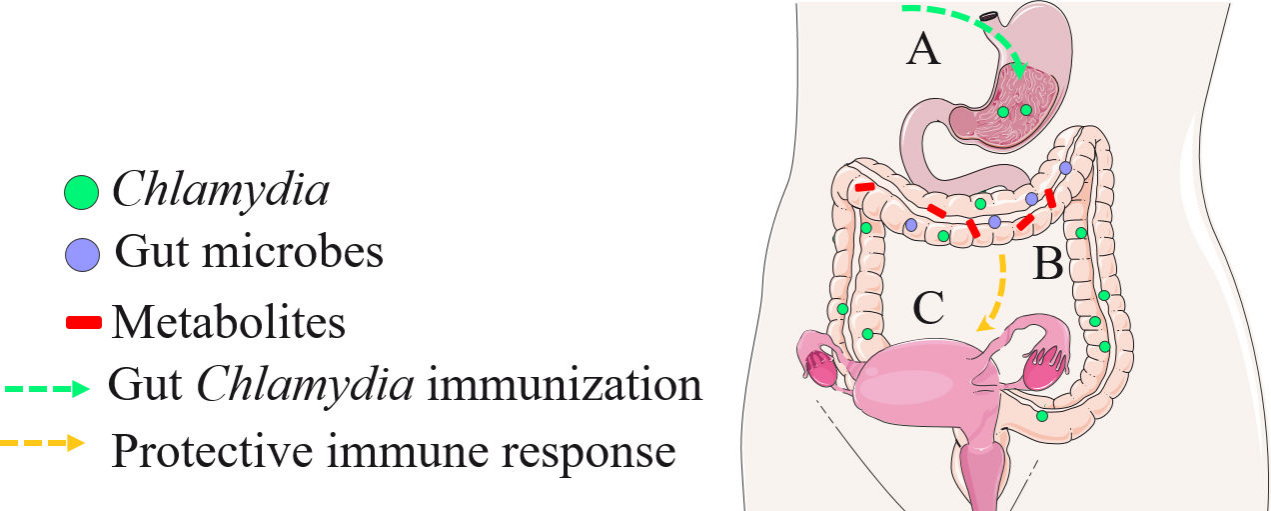
Schematic overview of the impact of oral *Chlamydia* vaccination on the gut microbiome, metabolite composition, and immune responses. The hypothetical interactions between oral *Chlamydia* vaccination, host gut microbiome, and metabolite composition. (**A**) Inoculation of *Chlamydia* into the GI tract leads to Chlamydial colonization within the GI environment. (**B**) Gut *Chlamydia* induces changes in microbial and metabolite profiles, which may regulate the host’s immune response against *Chlamydia*. (**C**) *Chlamydia*-specific transmucosal immune effects on the host are depicted. Portions of the figure utilize images from Servier Medical Art, licensed under the Creative Commons Attribution 3.0 Unported License.

The observed alterations in gut microbial diversity and metabolite composition following vaccination suggest that specific members of the gut microbiome and metabolites may serve as key mediators of vaccine efficacy (39). The identification of 129 distinct microbial taxa and 186 differentially abundant metabolites illustrates the complex biochemical environment influenced by *Chlamydia* vaccination, suggesting that these changes may affect the host’s immune responses against subsequent *Chlamydia* exposures (Fig. 6). Notably, certain genera, such as *Lactobacillus*, showed positive correlations with antibody production, indicating their potential as biomarkers for vaccine efficacy. Furthermore, we find that metabolites such as L-DOPA and PAF C-16 exhibited negative correlations with antibody levels, suggesting that these factors may lead to less favorable immune outcomes (Fig. 7). Considering L-DOPA is a precursor to the neurotransmitter dopamine and PAF C-16 is a signaling molecule that plays a role in inflammation and immune responses, both metabolites can be produced by gut microbes, and they may help to regulate mucosal immune responses in the gut by affecting inflammation and the activation of immune cells (40–42). Gut microbiota-derived metabolites, including dopamine, may also play a critical role in regulating immune cell functions, including Trm (15, 43). In the context of *Chlamydia* vaccine development, the generation and regulation of Trm cells are particularly important, as effective pathogen clearance relies on the reactivation of *Chlamydia*-specific Trm (6). Gut microbiota-derived metabolites influence Trm cells in various ways. For example, they promote the conversion of vitamin A into retinoic acid, which enhances the expression of the chemokine receptor CCR9 and the integrin α4β7 (43). These molecules are essential for directing T cells to the intestine and supporting Trm cell development (44, 45). Additionally, microbiota-induced immune signals have been shown to promote Trm cell survival and modulate inflammation (46–48). While our study did not directly measure Trm cells or cytokine levels, the findings support linking gut microbiota diversity to immune responses and underscore the importance of evaluating vaccine efficacy in individuals with various microbiome compositions (46).

Another interesting observation is that, although live *Chlamydia* was consistently recovered from the gut via rectal swabs in the live *Chlamydia* group (Fig. 1A), it was only detectable on day 7 through 16S sequencing when rectal shedding levels were elevated (Fig. 4C). As an intracellular pathogen, *Chlamydia* primarily resides within infected epithelial cells, and fecal samples usually contain fewer shedding cells compared to rectal swabs. This suggests that feces may not be the optimal sample for detecting gut *Chlamydia* infections (11).

Despite these insights, this study has limitations that should be acknowledged. First, while we focused on gut microbiome and metabolite analysis, we did not conduct comprehensive serological metabolomics, which limits our understanding of systemic metabolic changes and their potential correlations with the gut environment and immune responses. Inclusion of serum metabolite profiling could provide a more complete picture of how oral vaccination influences host metabolism. Furthermore, although we identified several potential biomarkers associated with vaccination efficacy, our findings are primarily rooted in observed changes in gut microbiota, metabolite alterations, and subsequent mathematical analyses. Further validation through dedicated animal studies will be essential to confirm these biomarkers and establish their biological relevance.

In summary, this study provides information for the potential interplay between oral *Chlamydia* vaccination, gut epithelium, gut microbiome, and gut metabolite profiles. The implications extend beyond *Chlamydia* vaccination alone, and the nature of oral live *Chlamydia* vaccination enables prolonged colonization in the gut, yielding important insights into *Chlamydia* persistence in this niche. This research enhances our understanding of the biological significance of gut colonization and improves our understanding of host*–Chlamydia* interactions. By elucidating these dynamics, our study may inform strategies for managing *Chlamydia* gut infections and the development of more effective approaches for detecting and preventing these infections in the future (3).

## MATERIALS AND METHODS

### Animals

C57BL/6J female mice, aged 5–6 weeks, were sourced from Vital River in Beijing and maintained under controlled environmental conditions (temperature: 22°C, light/dark cycle: 12 hours each). The mice were randomly assigned to three groups: control, heat-inactivated *Chlamydia*, and *Chlamydia*. For individual sample labeling during the experiment, C denotes samples from the control group; D denotes samples from the deactivated *Chlamydia* group; and L denotes samples from the live *Chlamydia* group.

### Preparation of chlamydial organisms

All *C. muridarum* clones utilized in this study were of the Nigg3 strain. The luciferase-expressing *C. muridarum* clone (G5-pGFP-Luci) has been previously documented (47). *C. muridarum* organisms were grown in HeLa cells, and density gradient centrifugation was used to purify EBs, as previously published (48). These purified EBs were aliquoted and stored at −80°C until required. The heat-inactivated *Chlamydia muridarium* was prepared by heating 2 × 10^6^ IFU of purified elementary bodies in Eppendorf tubes at 56°C water bath for 30 minutes, and complete inactivation was confirmed by infecting fresh HeLa cells with the heat-treated EBs for 24 hours and observing the absence of chlamydial inclusions through immunohistochemistry under a microscope.

### Immunization, challenge infections, and assessment of pathology

We followed the basic protocols and evaluation system of *Chlamydia* vaccinations previously published by Guangming Zhong (UT Health San Antonio). In short, to immunize the mice, purified *C. muridarum* EBs were intragastrically administered to female mice aged 6–7 weeks, reflecting the procedures described previously (38). Each mouse received an inoculation of 2 × 10^5^ IFUs of either live or deactivated *C. muridarum*, mimicking oral immunization. The control group was administered SPG alone, and the experimental groups received live or deactivated EBs diluted in SPG. Following each inoculation, vaginal and rectal swabs were collected periodically to monitor the colonization of viable *C. muridarum* in the gut and genital tract. Additionally, fecal samples from each group were preserved in liquid nitrogen for further experiments, and serum samples were collected at various time points.

Fifty days post-immunization, the remaining mice were challenged intravaginally with 2 × 10^5^ IFUs of *C. muridarum*, after which shedding from the genital tract and gut was monitored through swabs, and the mice were sacrificed on day 60 post-challenge for evaluation of genital pathologies with a focus on hydrosalpinx in the upper genital tract, utilizing high-resolution digital photography to document the oviduct hydrosalpinx, which was subsequently scored for severity and incidence within each group (9).

To assess the effects of immunization on colon epithelial cells, a separate cohort of mice was utilized. On days 7, 14, 30, and 60 post-immunization, three mice from each treatment group were euthanized for evaluation. Both H&E staining and TEM were conducted on the collected colonic tissue samples as previously published (49, 50). All observed pathologies were independently evaluated by two blinded pathologists to ensure accuracy and objectivity.

### Monitoring of *Chlamydia* infections

To monitor the shedding of live organisms, cervicovaginal and anorectal swabs were collected every 3–4 days for the first week, followed by weekly collections. Each swab was immersed in 0.5 mL of SPG and vortexed with glass beads to extract chlamydial organisms, which were then titrated on HeLa cell monolayers in duplicate. Inclusions were counted in five random fields per coverslip under a fluorescence microscope. For coverslips with fewer than one IFU per field, the entire coverslip was counted. Coverslips exhibiting significant cytotoxicity in HeLa cells were excluded from analysis. The total number of IFUs per swab was calculated based on the average IFUs per view, the area ratio of the view to that of the well, dilution factors, and inoculation volumes. If applicable, mean IFUs per swab were derived from serially diluted and duplicate samples. The total count of IFUs per swab was converted to log10 and used to compute the mean and standard deviation for the mice within each group at each time point.

For live imaging, mice infected with the luciferase-expressing *C. muridarum* clone (G5-pGFP-Luci) were imaged using the Xenogen IVIS imaging system (PerkinElmer, Hopkinton, MA, USA) on various days after infection. Prior to imaging, 500 µL of D-luciferin (40 mg/mL in sterile phosphate-buffered saline [PBS]) was injected intraperitoneally into each mouse. Twenty-five minutes after injection, the mice were anesthetized with 2% isoflurane. Bioluminescent images of the entire mouse were captured as described previously (51).

### ELISA for measurement of mouse fecal IgA and serum IgG antibodies

To assess IgA antibodies, fecal samples from each mouse were resuspended in PBS solution to achieve a final concentration of 1 mg/10 µL. After centrifugation at 13,000 rpm for 5 minutes, the supernatants were applied neat or diluted twofold to 96-well plates precoated with purified *C. muridarum* EBs. IgA binding was detected using goat antimouse IgA conjugated with horseradish peroxidase (HRP; catalog number 626720; Invitrogen, Waltham, MA, USA) along with a soluble substrate, 2,2′-azinobis(3-ethylbenzothiazoline-6-sulfonic acid) diammonium salt (catalog number 30931670; Sigma-Aldrich, St. Louis, MO, USA). Absorbance readings were taken at 405 nm using a Synergy H4 microplate reader (BioTek, Winooski, VT, USA), with results expressed as raw optical density (OD) values. For IgG detection, serum samples collected from the tail vein of mice were subjected to a fourfold serial dilution starting at 1:1,600. IgG binding to plate-coated EBs was identified using a goat antimouse IgG–HRP conjugate (catalog number 31430; Thermo Fisher Scientific, USA) as previously described. The same ELISA protocol was applied for IgG isotyping, with serum samples diluted 1:1,600 on *C. muridarum*-coated plates. Results were also reported as raw OD values.

### DNA extraction and 16s rRNA gene amplicon sequencing

Genomic DNA extraction was performed using the OMEGA Soil DNA Kit (M5635-02; Omega Bio-Tek, Norcross, GA, USA), in accordance with the manufacturer’s instructions, and samples were stored at −20°C prior to analysis. The quality and quantity of the extracted DNA were evaluated using a NanoDrop NC2000 spectrophotometer (Thermo Fisher Scientific) and agarose gel electrophoresis, respectively.

PCR amplification of the V3–V4 region of bacterial 16S rRNA genes was conducted using the forward primer 338F (5′-ACTCCTACGGGAGGCAGCA-3′) and reverse primer 806R (5′-GGACTACHVGGGTWTCTAAT-3′). Sample-specific 7 bp barcodes were included in the primers for multiplex sequencing. Each PCR reaction contained 5 µL of 5× buffer, 0.25 µL of Fast Pfu DNA Polymerase (5 U/µL), 2 µL of 2.5 mM dNTPs, 1 µL (10 µM) of each primer, 1 µL of DNA template, and 14.75 µL of ddH_2_O. The thermal cycling protocol included an initial denaturation at 98°C for 5 minutes, followed by 25 cycles of denaturation (98°C for 30 s), annealing (53°C for 30 s), and extension (72°C for 45 s), capped with a final extension at 72°C for 5 minutes. PCR amplicons were purified using Vazyme VAHTS DNA Clean Beads (Vazyme, Nanjing, China) and quantified with the Quant-iT PicoGreen dsDNA Assay Kit (Invitrogen, Carlsbad, CA, USA). Following quantification, amplicons were pooled in equal concentrations, and paired-end 2 × 250 bp sequencing was executed on the Illumina NovaSeq platform with the NovaSeq 6000 SP Reagent Kit (500 cycles) at Suzhou PANOMIX Biomedical Tech Co., Ltd. Sequence data analyses were primarily completed using QIIME2 and R packages (v.4.4.2) with default parameters. The analysis results for 16S rRNA sequencing were visualized by R package MicrobiotaProcess (v.1.20.1). α-Diversity indices at the ASV level, such as Chao1 richness estimator, observed species, Shannon diversity index, and Simpson index, were calculated from the ASV table in QIIME2 and visualized as box plots (Fig. S1). ASV-level ranked abundance curves were generated to assess richness and evenness across samples. β-Diversity analyses examined the structural variation of microbial communities among samples, and the correlation among samples was analyzed (Fig. S2) using Bray–Curtis and UniFrac distance metrics, with results visualized through principal coordinate analysis. Principal component analysis was also performed on genus-level compositional profiles. Taxonomic compositions and abundances were visualized, and LEfSe was applied to identify differentially abundant taxa across groups. Clustering tree analysis of various samples is shown in Fig. S3.

### LC-MS/MS analysis

LC analysis was performed using a Vanquish UHPLC System (Thermo Fisher Scientific) by Suzhou PANOMIX Biomedical. Chromatography was conducted with an ACQUITY UPLC HSS T3 column (2.1 × 100 mm, 1.8 µm; Waters, Milford, MA, USA) in both positive and negative ion modes (52). The Orbitrap Exploris 120 mass spectrometer (Thermo Fisher Scientific) was utilized for the detection of metabolites, featuring an electrospray ionization (ESI) ion source and operating in data-dependent acquisition mode. The instrument was configured with a sheath gas pressure of 40 arb, an auxiliary gas flow of 10 arb, and spray voltages set at 3.50 kV for positive ionization and −2.50 kV for negative ionization, maintaining a capillary temperature of 325°C, with a mass range of *m*/*z* 100–1,000, an MS1 resolving power of 60,000 FWHM, and an MS/MS resolving power of 15,000 (53), executing four data-dependent scans per cycle. The Mfuzz method was employed for cluster analysis of metabolite patterns across different samples, utilizing a fuzzy c-means algorithm to establish relationships between time and metabolites across clusters (54).

### Statistical analysis

Quantitative data, including viable organism counts (IFUs) and genome copies, were analyzed using the area under the curve method, the Wilcoxon rank-sum test, and the Kruskal–Wallis test. For qualitative data, such as incidence rates, Fisher’s exact test was employed. Bonferroni corrections were used to adjust the significance level for each individual test for multiple comparisons. Additionally, semiquantitative data were also analyzed using the Wilcoxon rank-sum test. A significance threshold of *P* < 0.05 was set to determine statistical significance.

## Data Availability

The *Chlamydia muridarum* clones utilized in this study were obtained from the Nigg3 strain (GenBank accession number CP009760.1). The microbiome data were deposited in the NCBI Sequence Read Archive (project number PRJNA1276300). The metabolomics data were deposited in Metabolights (ID: MTBLS12603). The original contributions presented in the study are included in the article and supplemental material; further inquiries can be directed to the corresponding authors.

## SUPPLEMENTAL MATERIAL

Figure S1 msystems.01285-25-s0001.tiff The dilution curves of the sequencing.

Figure S2 msystems.01285-25-s0002.tiff Temporal changes in gut microbiota composition and abundance at the phylum and genus levels.

Figure S3 msystems.01285-25-s0003.tiff β-Diversity analysis of gut microbial communities among treatment groups over time.

Figure S4 msystems.01285-25-s0004.tiff Microbes with linear discriminant analysis scores greater than 2.

Figure S5 msystems.01285-25-s0005.tiff KEGG pathway analysis of the metabolites with KEGG IDs.

Legends msystems.01285-25-s0006.docx Legends for the supplemental material.

Table S1 msystems.01285-25-s0007.xlsx Metabolite first and second level identification.

Table S2 msystems.01285-25-s0008.xlsx Significantly different metabolites.

ASM does not own the copyrights to Supplemental Material that may be linked to, or accessed through, an article. The authors have granted ASM a non-exclusive, world-wide license to publish the Supplemental Material files. Please contact the corresponding author directly for reuse.
