# Supplementary figures and images for: Impact of oral Chlamydia vaccination on host gut microbiome and metabolite composition
Source: mSystems. 2025 Nov 10;10(12):e01285-25. doi: 10.1128/msystems.01285-25 (PMC12710358; doi:10.1128/msystems.01285-25)

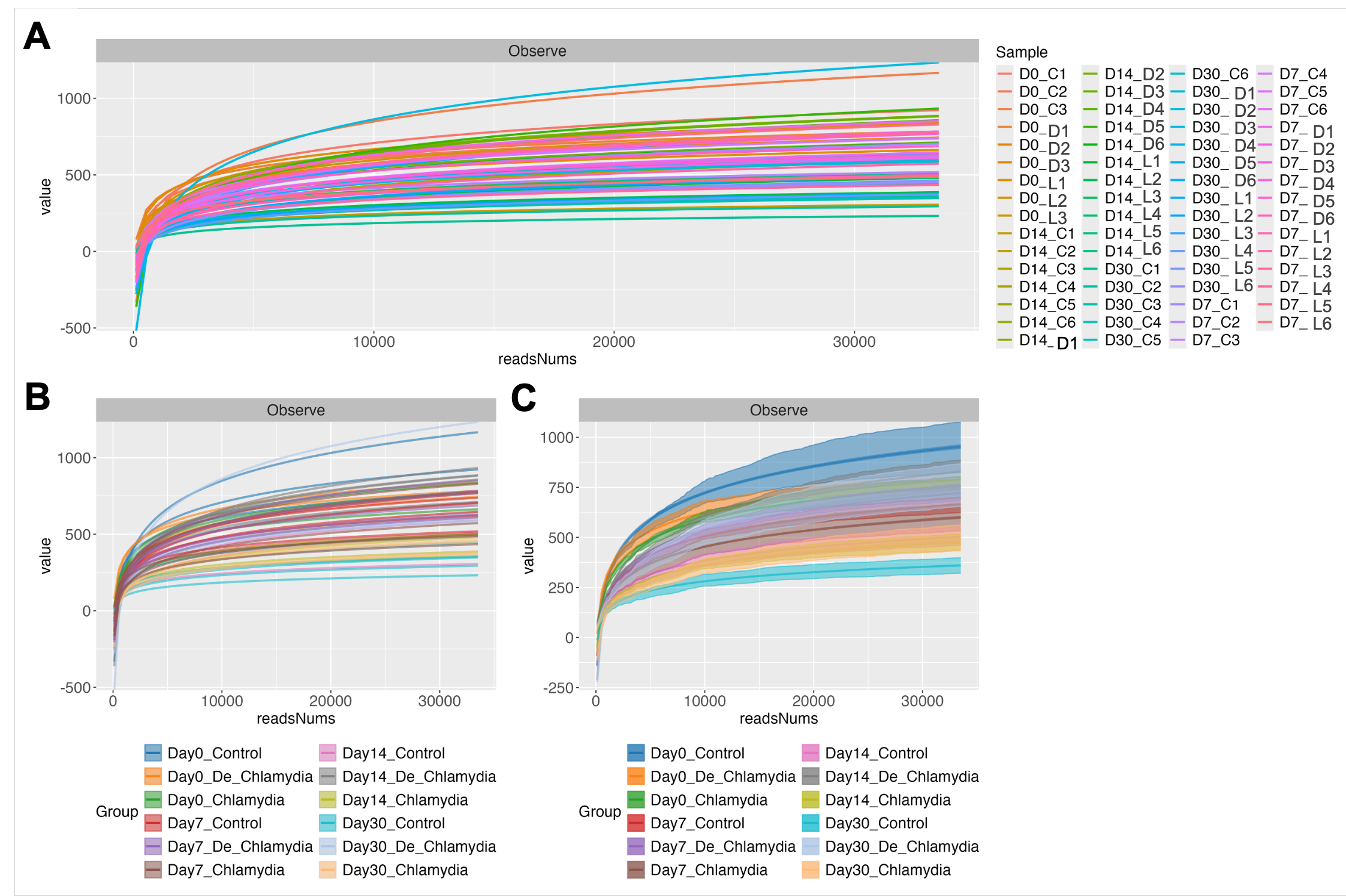

Supplement: Figure S1 — The dilution curves of the sequencing. [file msystems.01285-25-s0001.tiff]

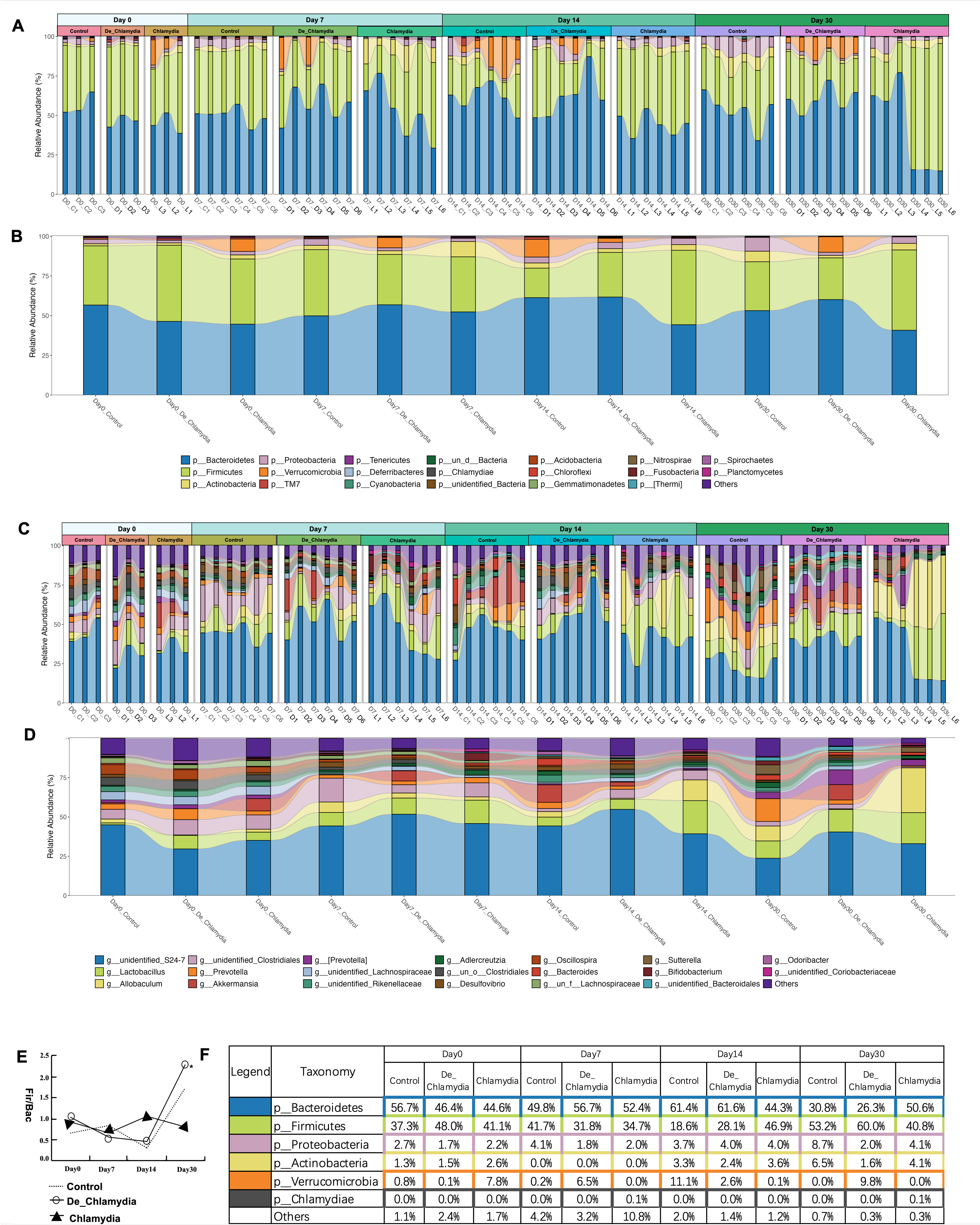

Supplement: Figure S2 — Temporal changes in gut microbiota composition and abundance at the phylum and genus levels. [file msystems.01285-25-s0002.tiff]

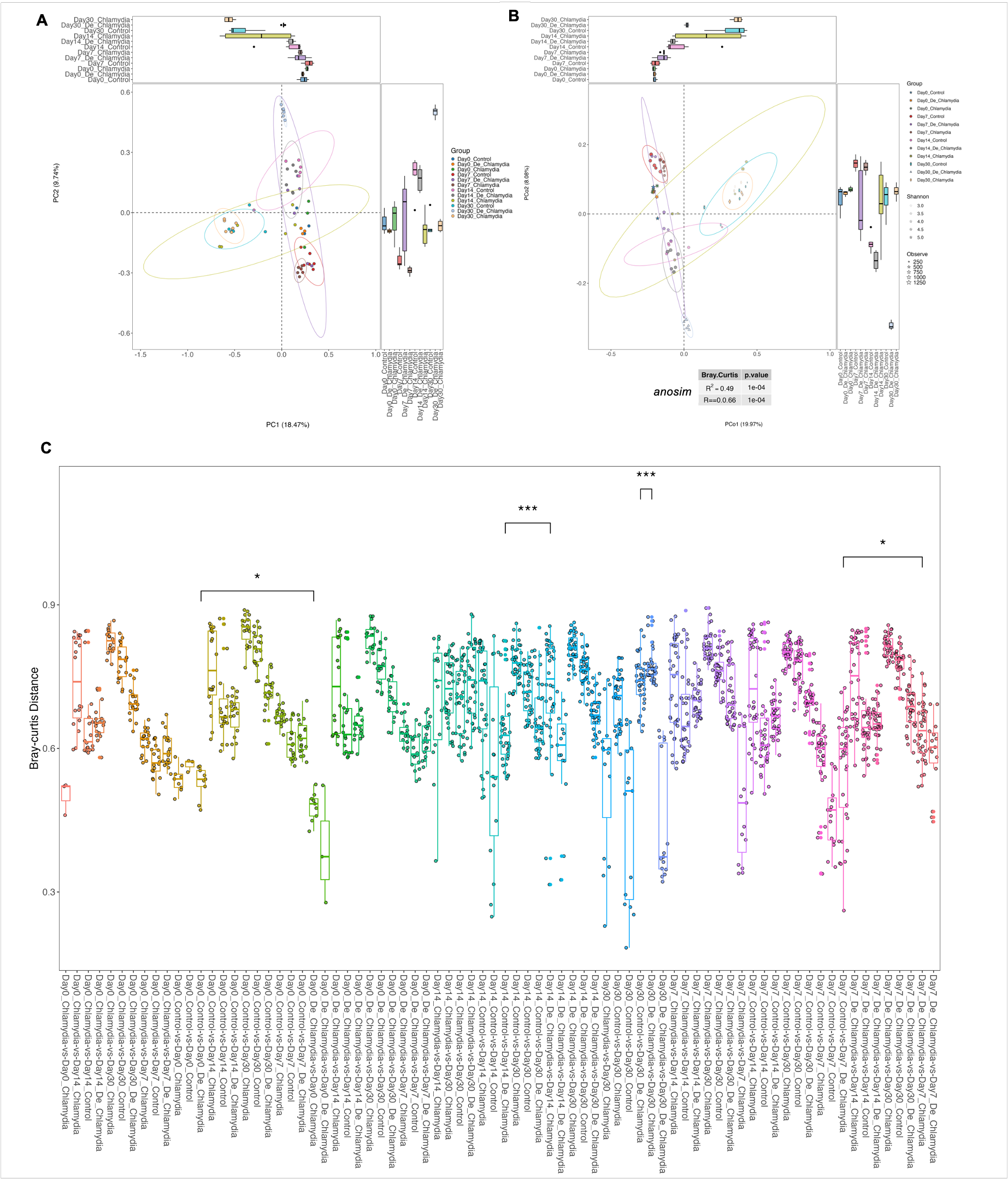

Supplement: Figure S3 — β-Diversity analysis of gut microbial communities among treatment groups over time. [file msystems.01285-25-s0003.tiff]

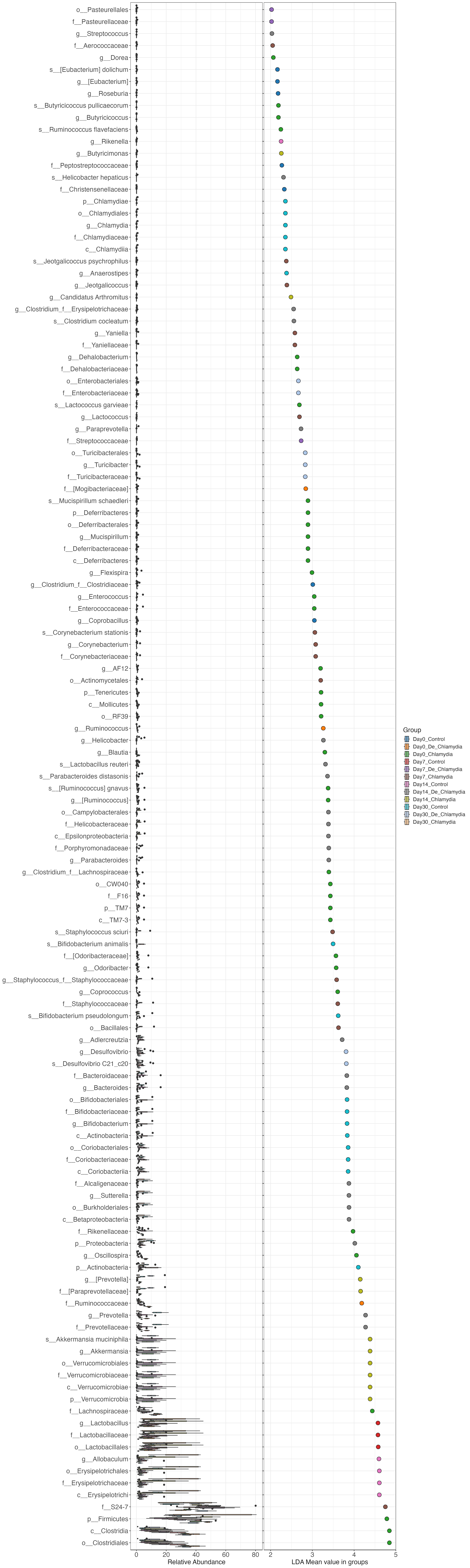

Supplement: Figure S4 — Microbes with linear discriminant analysis scores greater than 2. [file msystems.01285-25-s0004.tiff]

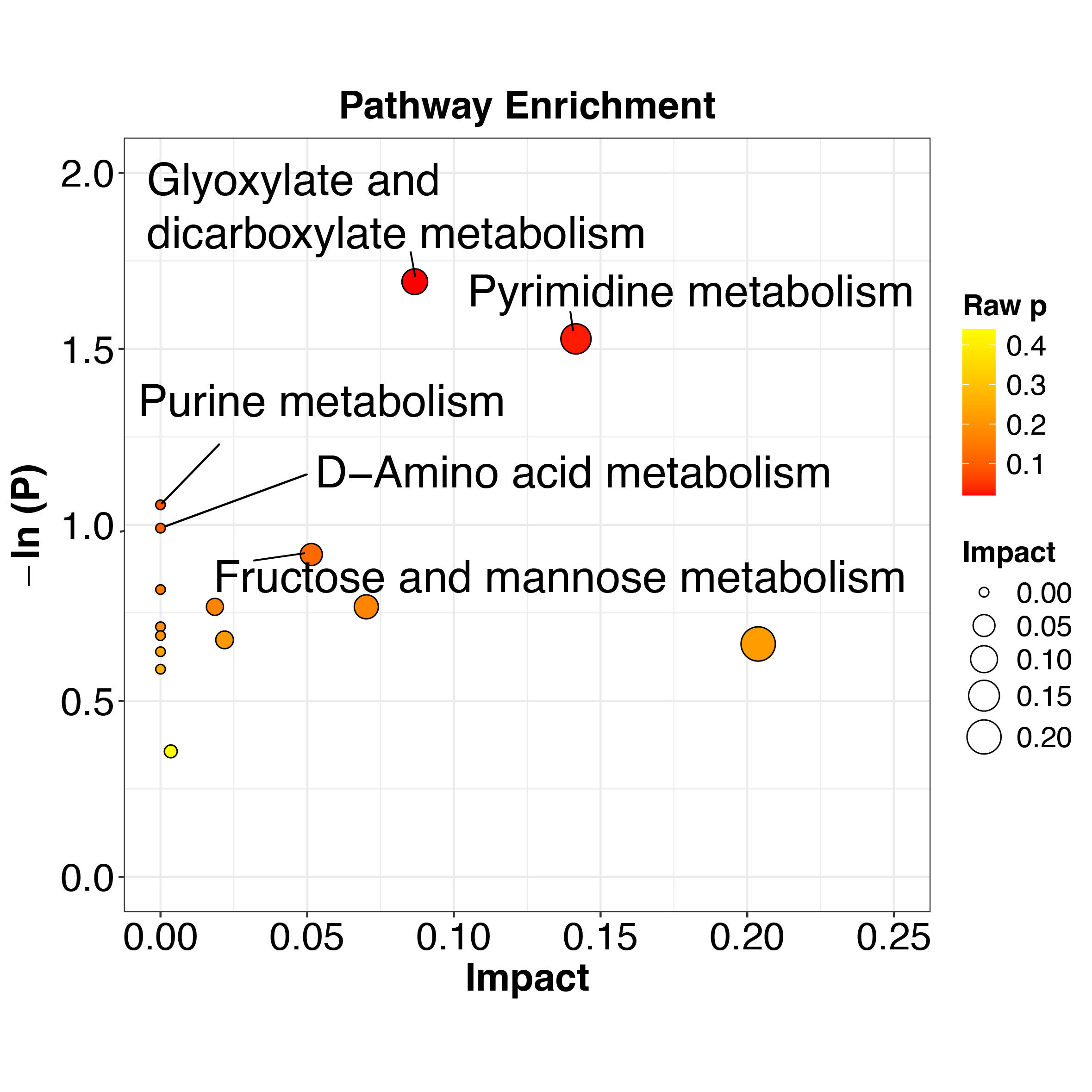

Supplement: Figure S5 — KEGG pathway analysis of the metabolites with KEGG IDs. [file msystems.01285-25-s0005.tiff]
