# Supplementary material for: Impact of oral Chlamydia vaccination on host gut microbiome and metabolite composition
Source: mSystems. 2025 Nov 10;10(12):e01285-25. doi: 10.1128/msystems.01285-25 (PMC12710358; doi:10.1128/msystems.01285-25)
Supplement: Legends — for the supplemental material. [file msystems.01285-25-s0006.docx]

**Supplementary Figure1 Sequencing dilution curves.**

Panels A–C show dilution curves for various samples and treatment groups, indicating that adequate sequencing depth was achieved. The x-axis represents the number of reads, with a plateau suggesting sufficient coverage. Panel A shows curves for individual samples; Panel B presents curves grouped by treatment and time point; Panel C displays the grouped curves with 95% confidence intervals. These results indicate that the majority of intestinal microbial diversity was captured and the sequencing depth was sufficient to represent gut microbiota diversity accurately.

**Supplementary Figure2:** **Temporal changes in gut microbiota composition and abundance at the phyla and genus levels.** Panels A and B illustrate the top 20 dominant phyla and their fluctuations across samples and treatment groups over the time series. Panels C and D highlight the top 20 changes in specific genera among the samples and combined groups. Panel E presents the Firmicutes to Bacteroidetes (Fir/Bac) ratio among the groups throughout the time series. Finally, Panel F features a table detailing the top five dominant phyla and the detection of *Chlamydia* during the time series across the different groups by 16S rRNA sequencing.

**Supplementary Figure3: β-diversity analysis of gut microbial communities among treatment groups over time.** Panel A illustrates Principal Component Analysis (PCA), an unsupervised dimensionality reduction method and Panel B presents Principal Coordinates Analysis (PCoA), a supervised dimensionality reduction method that identifies distinct clustering of microbial communities across groups. The Bray-Curtis dissimilarity is utilized to evaluate the compositional differences in species across groups (Panel C), based on quantitative characteristics of the species present in the samples (**p* < 0.05, ****p* < 0.001)

**Supplementary Figure4:**

Microbes with linear discriminant analysis scores greater than 2.

**Supplementary Figure5:**

KEGG pathway analysis of the metabolites with KEGG IDs (The top 5 P value enriched metabolic pathways have been labeled).

**Supplementary table 1**

The summary of first and second level metabolites identified.

**Supplementary table 2**

The table of significant different metabolites (*p<0.05*) between time points.
